# From bench to bedside: the realities of reducing global prostate cancer disparity in black men

**DOI:** 10.3332/ecancer.2014.458

**Published:** 2014-08-28

**Authors:** Robin Roberts

**Affiliations:** The University of the West Indies School of Clinical Medicine and Research, Princess Margaret Hospital, Shirley Street, P. O. Box GT-2590, Nassau, Bahamas

**Keywords:** prostate-specific antigen, prostate cancer biomarkers, baseline PSA, prostate cancer screening, prostate cancer incidence, prostate cancer risk, African American, black men, African Caribbean, epigenetics in prostate cancer, castration-resistant prostate cancer, prostate cancer disparity, bench-to-bedside, global prostate cancer, culture and health-seeking behaviours, homophobia

## Abstract

Prostate cancer in black men of African descent has a different tumour biology compared to those of other races. Its clinical manifestations depict a more aggressive disease with higher morbidity and mortality. This study proposes, through a literature search, identifying applied laboratory and clinical research in prostate cancer directed to improve outcomes and decrease global disparities of prostate cancer in black men of African descent. This review identified five categories pertinent for research: prostate-specific antigen (PSA) testing for early detection and screening, the potential of epigenetics, cultural determinants and health-seeking behaviours, other biomarkers for prostate cancers, and the economics of treating advanced prostate cancer. The analysis revealed that in developed countries, men of African descent are underrepresented in the sampling pools in both laboratory and clinical research, and thus the applicability and relevance of these results to men of African descent are circumspect. However, developing countries with high populations of black males have limited laboratory and clinical research publications. This is due to limited funding to support research programmes and basic clinical services for early detection and treatment. The study concludes that for the involvement of developing countries in bench research, they should do it in collaboration, like fostering partnerships with credible academic-based institutions and organisations. This requires a realm of transparency, respect, protection of the rights and dignity of the patients, and an equity in participation and sharing of the benefits to be accrued. The current transatlantic and Caribbean collaborations in research, education, and health service delivery in prostate cancer care for men of African descent exemplify the successes of such partnerships.

## Introduction

Prostate cancer is the most common cancer in men worldwide and one of the leading causes of death from malignancies [1]. This is a global phenomenon with an estimated 258,000 deaths annually [1]. With a one in seven probability of being diagnosed with prostate cancer over a lifetime [2] and the media attention given to celebrities being diagnosed and treated for prostate cancer, men cannot evade the inevitability of aging and facing their vulnerability as they age.

The issues arising in both the diagnosis and treatment of prostate cancer are controversial: Who and when to get tested? Which tests? What treatment, if any? There are no shortage of guidelines, options, and recommendations expounded by national government agencies, non-governmental, and professional organisations, both acting as lobbyists and advocates, often with varying interests and opposing views. Finally, it becomes a logistical nightmare for men and their families to manoeuvre through this labyrinth [3–10].

Translational science has been a central force in advancing prostate cancer care and control, and is likely to contribute to the understanding of global prostate cancer disparities in black men. In line with the goal of the Second Biennial Science of Global Prostate Cancer Disparities in Black Men Conference [11], which is to explore, analyse, and minimise the current disparities in prostate cancer care, this paper seeks to identify applied laboratory and clinical research in prostate cancer directed to improve outcomes and decrease global disparities in black men of African descent.

## Methodology

A qualitative review of the literature was conducted. This included a systematic search of the PubMed database for articles using the keywords: ‘prostate specific antigen’, ‘prostate cancer biomarkers’, ‘baseline PSA’, ‘prostate cancer screening’, ‘prostate cancer incidence’, ‘prostate cancer risk’, ‘African American’, ‘black men’, ‘African Caribbean’, ‘epigenetics in prostate cancer’, ‘castration resistant prostate cancer’, ‘prostate cancer disparity’, ‘bench-to-bedside’, ‘global prostate cancer’. Original full-text articles published in English were reviewed. The reference lists of identified articles were searched for further relevant papers. In addition, a Google search was undertaken for the following keywords: ‘culture and health seeking behaviours’ and ‘homophobia’. No limits were set on the years of publication. A special emphasis was directed to the unique challenges facing global disparities and cultural associations.

## Results and discussion

### PSA testing beyond the bedside: a yea or nay, or just a matter of time?

Huggins’ Nobel Prize research on the response of prostate cancer to androgen ablation was a landmark event in the treatment of prostate cancer [12]. The discovery of the prostate-specific antigen (PSA) in 1970 and its US Food and Drug Administration (FDA) approval for monitoring prostate cancer recurrence in 1986 and for early diagnosis of prostate cancer in 1994 [13] were watershed moments in prostate cancer management. PSA screening heralded the exponential increase in the detection of prostate cancer in the 1990s [14, 15]. Due to the clinical evidence and value supporting early diagnosis of prostate cancer, it highlighted a 40% decrease in mortality rates in the 1990s, and with a 75% decrease in patients presenting with metastatic disease on initial presentation [16]. Stage migration, the shift of late-stage to early-stage disease at the time of diagnosis, became the hallmark and surrogate of PSA population-based testing for effective early detection programmes. PSA testing may even be considered a benchmark for cancer care in men; early detection and early stage disease led to interventions with better outcomes as more evidence emerged to favour radical prostatectomies for those under 65 years of age [17]. These advances in prostate cancer care in the PSA era, however, do not translate necessarily into a cause and effect scenario.

Major challenges are being mounted to counteract the merits and value of PSA testing in the diagnosis of prostate cancer [4]. Central to the debate is the well-documented natural history of this disease, that all men if they live long enough will have histological evidence of prostate cancer though most of them will have no clinical manifestations of this disease in their lifetime. Hence, it is logical to deduce that many men are being treated unnecessarily. The United States Prevention Task Force (USPTF) concluded that interventions to treat these insignificant non-progressing malignancies, detected solely by an elevated PSA test, have resulted in unacceptable morbidities in both diagnosis and treatment, with issues of severely impaired quality of life. But more importantly, the USPTF held that PSA testing does not decrease survival, the sine qua non of a screening test. In noting the high morbidity and commercialisation of PSA testing, Richard Ablin, a pioneer and proponent of PSA testing for recurrent prostate detection only, called his discovery the ‘Great Prostate Mistake’ [18]. Blum and Scholz wrote a book against this early detection of low-risk disease, and the aggressive treatment and morbidities of unnecessary interventions: ‘The Invasion of the Prostate Snatchers’ [19]. Laurence Stains gave testimonial that ‘I want my prostate back’ [20]. Thomas Stamey, one of the gurus and advocates of PSA screening in the early 1990s, declared that ‘the PSA era is over’ as he questioned the value of PSA in the detection of prostate cancer as opposed to simply benign prostatic hypertrophy (BPH) [21].

The debate on screening, and whether it decreases mortality rates or not, remains unresolved. The long-awaited prospective randomised transatlantic trials were inconclusive and only heightened the debate [22–25]. While the Goteborg study [24] and the extended trial at 11 years follow-up in the European study [26], documented significantly decreased mortality from the screening initiative as in its initial report two years earlier, in the extended follow-up in the American-based trial, a decrease in mortality remained of no statistical significance [27].

Unfortunately, the PSA era has bypassed men of African ancestry. Representation of black men in PSA research has been miniscule at best, as noted in the Prostate, Lung, Colorectal, Ovarian (PLCO) trial: ‘Blacks and Hispanics were nonetheless underrepresented in PLCO compared to their levels among age-eligibles in the overall US population or in the aggregate PLCO catchment areas’ [28]. The recommendations for PSA screening in black men are guided more by the aggressiveness of the disease, the morbidity and mortality rates, the economics of health-care delivery, access, and availability health-care services. Research on black male populations specifically for PSA baselines, longitudinal studies, and negative and positive predictive values for determined PSA levels are lacking. At the sunset of the PSA era, the USPTF recommends against PSA-based screening, for all men of all ages. The statement recognised that black men have stronger family histories and are more likely to die of the disease, but indicated that:

No firm conclusions can be made about the balance of benefits and harms of PSA-based screening in this population. However, it is problematic to selectively recommend PSA-based screening for black men in the absence of data that support a more favourable balance of risks and benefits. A higher incidence of cancer will result in more diagnoses and treatments, but the increase may not be accompanied by a larger absolute reduction in mortality [29].

In essence, PSA is not the biomarker to invest in, to advance the care of prostate cancer in men of African descent. The statement does not lend for more research investment in PSA testing and black men, especially with no clinical manifestations of prostate cancer.

## The global disparity of prostate cancer mortality

The merits of PSA in the transition from the ‘bench to the bedside’ cannot be denied. Earlier disease presentations have led to better treatment modalities and outcomes as well as increased long-term survival rates [16]. More impressive is the early-stage presentation of prostate cancer: it has gone from a 20% initial early disease presentation to an 80% early clinical disease at diagnosis [28–31]. This stage migration has yet to occur in developing countries [32]; it correlates directly with the lack of PSA screening, access and availability of health-care services, and health prevention and promotion programmes in developing countries.

In men of African descent, worldwide, the mortality rates are much higher compared to those of men of other ethnicities [33]. Data from the Surveillance, Epidemiology, and End Results (SEER) Programme clearly indicate a higher incidence in African Americans, both in terms of morbidity and mortality at an earlier age, and with more advanced disease at initial onset [34]. The high mortality rates in the Caribbean led the global trends data [1]. The estimated number of prostate cancer deaths in Sub-Saharan Africa during 2008 was more than five times that of African Americans and is expected to double in Africa by 2030 [33].

It is interesting to note that the Detroit Education and Early Detection Study revealed that PSA screening could equalise the early detection and organ-confined disease in white and black Americans with equal access and availability of health-care services [35]. Follow-up studies indicated that with continued early detection, PSA driven initiatives, and equal health-care services, both organ-confined disease and survival outcomes between the two ethnicities showed no significant differences [36].

British men of Caribbean and African descent, while having higher risk of prostate cancer and earlier age of diagnosis, appeared to have no difference in mortality under the universal health-care service in the UK [37]. PSA screening in Britain is not practised; it would be interesting to speculate that with PSA screening there may have been no difference in risk of occurrence or age of onset, as concluded in the Detroit study.

A country profile for prostate cancer in the Bahamas reveals the absence of, and hence the need for, PSA screening in a community of men of African descent where prostate cancer is at high risk [32]. Over the past 20 years, the incidence of men presenting with advanced prostate cancer disease on initial presentation has remained unchanged as in the pre-PSA era. Men of African descent must be an exception to the USPTF directive. Developing countries with limited resources must aggressively introduce and continue national PSA screening initiatives if they are to reduce the burden of metastatic prostate cancer and also this global prostate cancer disparity [38, 39].

The high burden of advanced disease due to late presentation compels for population-based programmes using PSA for early detection to reduce the high morbidity and mortality occurring with the late clinical presentation. It is only when this stage-shift migration occurs, that a community can have the luxury to debate the value of PSA in the early diagnosis of prostate cancer. Thus the research and debate in the post PSA era should not be on its utility or to devalue its contributions, but should be rather to focus on improving treatment selection and to identify the factors or biomarkers to define indolent versus aggressive cancers to influence treatment options.

### Bench research in the behavioural research laboratory

An important question to be answered is this: Is the lack of the acceptance of PSA screening in developing countries primarily a resource issue? There is much to suggest that the ‘bench research’ is yet to be done in developing counteries. The bench research is not with test tubes, chemicals, and pipettes, but research embedded in the Behavioural Research Laboratory, investigating human decision-making and behaviour.

'What does culture have to do with it' [41]? Culture may have a major impact on health-seeking behaviours in men of African descent and at times impedes men from availing themselves of the ready access and availability of prostate cancer screening. This study of 335 African American and Bahamian males, both of African descent, revealed a strong association between cultural health values, beliefs, practices, risk for illness and disease, and what for them constitutes a healthy state; these were seminal factors affecting health-seeking behaviours. The macho male culture may have a strong negative influence on the willingness of the African male to undergo PSA testing and rectal examination for prostate cancer detection. ‘Real Men’ behaviour is displayed as a badge of honor in the community. Refusal of annual checkups, denial of symptoms of illness or pain, and the undertaking of risk-seeking behaviours: these all are key elements of the ‘Real Men’ construct [26].

A refusal to have a rectal examination is a vital component of this social persona. Media headlines suggest that this refusal of the digital rectal examination underscores a more fundamental behavioural trait, namely, homophobia. Is it coincidental that Jamaica, cited on one hand as having the highest prevalence of prostate cancer in the world [39], is noted to be the most homophobic place on earth [41]. Other Caribbean Islands and Africa have also been noted to have homophobic actions. For example, in 2004, angry protestors in the Bahamas met gay cruise ship passengers on the docks [43]. Homosexuality is illegal in many African countries—particularly Arab North Africa and those with a British colonial past such as Kenya, Uganda, and Malawi; in Uganda, parliamentarians have proposed not only a life sentence, but also the death penalty as well [44].

There are limited studies on human decision-making and behavior in men of African descent seeking early detection and treatment [45–47]. A considerable percentage of men of African descent afflicted with prostate cancer, indolent or clinical, remain bedridden [48]. What continues to influence them not to seek medical care even when they become symptomatic?

### Bench research: a focus on epigenetics

The new science of epigenetics focuses on the impact of dietary, lifestyle, cultural, and environmental factors on the initiation and progression of prostate cancer at the level of the genes.

Fat has been the focus of dietary studies of prostate cancer more than any other dietary component [47]. The emerging epidemic of obesity in developing countries [49] has grave implications for increasing prostate cancer prevalence and burden, and projects for even greater health-care disparity. Animal studies suggest that dietary fat increases both the incidence and the rate of growth of adenocarcinomas of the prostate in laboratory animals, and at the clinical level, the evidence suggests that high-fat diets focus at the level of converting indolent subclinical cancers to clinical ones [50]. Weight loss may reduce the risk of prostate cancer, and obesity increases the risk [51]. Lifestyle, obesity, metabolic syndrome, and diet no doubt share a complex relationship in prostate cancer biology. Certainly, epigenetics, and behavioural research are intertwined, but their bedside clinical applications are still pending.

Research continues to highlight and identify the process of prostate carcinogenesis [52]. The quest to identify tumour markers to determine indolent versus aggressive tumours remains ongoing as well [53]. The search for gene patterns for aggressive disease is still in its early stages too. Molecular mechanisms of prostate cancer initiation have produced a shopping list of novel biomarkers for castration-resistance, but are not clinically applicable [54]. In summary, biomarkers are sought that will lend to understanding the natural and complex biology of prostate cancer, and improving the management of cancer patients by enhancing the efficiency of detection and efficacy of treatment. The bench work is still a long way from clinical application.

### Biomarkers beyond PSA

In the early diagnosis catalog of potential biomarkers, the FDA has approved PCA3 gene detection in the urine. This shows promise in decreasing the false-positive rates for PSA monitoring and reducing the need for prostate biopsies. The test cannot be used as a free-standing test for detection and thus has limited clinical utility [55].

A new isoform of PSA, pro-PSA has also been approved by the FDA. It has the distinction of possibly being a better predictor for prostate cancer than the total or free PSA [56]. Concomitantly, the FDA approved the use of the ‘Prostate Health Index’ which combines measurements of total PSA, free PSA, and pro-PSA. Research suggests that the index is 2.5 times more specific in detecting prostate cancer in patients than PSA screening. This has the promise of reducing over-detection of indolent, less-aggressive prostate cancers, thus decreasing the indications for biopsies.

An even greater potential for early detection lies in the discovery of the allelic region of the 24th region of the 8th chromosome designated the 8q24 gene. In this ‘hot-spot’ region, alleles are differentially expressed in metastatic and non-metastatic prostate cancer cells. Men with prostate cancer who are carriers of genetic variants on chromosome 8q24 are more likely to have family members at higher risk for prostate cancer [57]. It is a more aggressive malignancy, and prostate cancer gene carriers are more prone to have higher Gleason scores, extracapsular tumour extension, seminal vesicle invasion, metastasis to lymph nodes, and overall worse cancer prognosis.

The pinnacle of early detection must be the potential to detect the risk of developing prostate cancer before the cancer gene mutation. The science of personal genomics, being able to sequence and analyse one’s genes, allows an individual to compare their genes with those identified for common diseases, including cancers. The possibility exists for individuals to identify a prostate cancer gene in their genome. This bench science was transplanted to the bedside with the Icelandic Health Sector Database project, a population-based study which sought to identify and register the DNA profile of all Icelanders, a fairly homogenous ethnic population of less than 300,000 people [58]. From this genomic database, allied with medical records, there was the potential to identify human genes associated with common diseases, including cancers. Through single nucleotide polymorphism, individuals could scan their own DNA to determine the possibility of these labelled common disease genes in their DNA profile. To be able to identify prostate cancer genes as early as birth allows for a lifestyle modification to minimise prostate cancer initiation.

While these new developments in biomarkers for prostate cancer are welcomed, their efficiency and applicability to black men are circumspect as the test samples of men of African descent in these sample populations are small. Moreover, these tests are expensive and costs prohibitive for use in developing countries.

## From bench to bedside: new drugs for advanced prostate cancers

From the therapeutic perspective, great strides have been made in new drugs for advanced prostate cancer, and in particular those that develop with hormonal resistance. Within the last four years, five novel agents, acting through distinct mechanisms, have been FDA approved for metastatic castration-resistant prostate cancer [59]. These new drugs are costly [60]: Cabazitaxel, $48,000 for a course of treatment; sipuleucel-T, $93,000; abiraterone, $5,000/month; and denosumab, $1650 per treatment per month. For developing countries, not only are these cost prohibitive, the outcomes—an increase of an average of four months median survival—lend very little value as these countries have limited resources. It is unlikely that these new drugs will ever be available to the population at large, and certainly will further increase rather than decrease the health-care disparity in prostate cancer care.

## Conclusion

Research translation from the bench to bedside is one in which developing countries’ focus must be collaborative. Scarce resources dictate that our research initiatives be directed to providing the vital clinical resources. This collaboration must be done within a realm of transparency, respect, and protection for the rights and dignity of the patients, and with an equity in participation and sharing in of the benefits to be accrued from the collaboration. Our coalitions to date have manifested these principles through the following consortia and organisations: Men of African Descent and Carcinoma of the Prostate Consortium, the Prostate Cancer Transatlantic Consortium, the Florida Cancer Disparity Group, the African Caribbean Cancer Consortium, and the African Organisation for Research & Training in Cancer. Our institutional partners, including the University of Florida, the National Institutes of Health, and the University of the West Indies, have been most supportive and generous with their collaborative assistance. It is through these partnerships, the realities of bench-to-bedside can be realised and brought to fruition for the benefits of our respective populations and thus, health-care disparities in prostate cancer care can be reduced for the betterment of all. Indeed, the whole is greater than the sum of the individual parts.

## Conflicts of interest

The author declares that he has no conflicts of interest.
